# Model‐supported decision‐making at a contaminated sediment site: Post‐audit and site closure

**DOI:** 10.1002/ieam.4556

**Published:** 2021-12-20

**Authors:** David Glaser, Kevin Russell, James Rhea, Wen Ku, Deirdre Reidy, Russell Cepko

**Affiliations:** ^1^ Anchor QEA, LLC Woodcliff Lake New Jersey USA; ^2^ Anchor QEA, LLC Liverpool New York USA; ^3^ ViacomCBS Inc. Pittsburgh Pennsylvania USA

**Keywords:** Bioaccumulation, Decision support, Fate and transport, Model, Post‐audit

## Abstract

Computer simulation models have been used to support decision‐making at contaminated sediment sites for decades. Nonetheless, their reliability in remedial decision‐making has been questioned, and there is a need for retrospective studies of the accuracy of model predictions, that is, post‐audits. The Neal's Landfill site near Bloomington, Indiana, provides an example of the successful use of a mathematical simulation model in the selection of a remedy for a site that includes streams with polychlorinated biphenyl (PCB)‐affected sediment, water, and fish. A chemical fate and transport and bioaccumulation computer simulation model was developed to compare the effectiveness of alternative remediation plans in reducing fish total PCB concentrations. A post‐audit of the model, using several years of data collected after remediation, demonstrates that the model successfully predicted declines in surface water and fish tissue PCB concentrations over a decade, including those associated with longer term natural recovery processes as well as the response to remedial actions. The model predicted, and the post‐audit bore out, that risk‐based goals would be met using an alternative less extensive than others under consideration. An uncertainty analysis, based on bounding model calculations, provided important support for decision‐making, as did the inclusion of a statistical Remedy Confirmation Clause in the Consent Decree for the site. This study demonstrates the utility of a computer simulation model to guide remedial decision‐making at a contaminated sediment site. *Integr Environ Assess Manag* 2022;18:1233–1245. © 2021 The Authors. *Integrated Environmental Assessment and Management* published by Wiley Periodicals LLC on behalf of Society of Environmental Toxicology & Chemistry (SETAC).

## Key Points

- The Neal's Landfill site provides an example of the successful use of a chemical fate and transport and bioaccumulation computer simulation model in selecting a remedy for a PCB‐affected stream.
- The model, including an uncertainty analysis based on bounding model calculations, provided important support for decision‐making.
- A post‐audit demonstrated that the model successfully predicted that risk‐based goals would be met using an alternative less extensive than others under consideration.

## INTRODUCTION

Computer simulation models have been used to support decision‐making at contaminated sediment sites for decades. The US Environmental Protection Agency (USEPA) recognizes models that incorporate hydrodynamics, sediment transport, chemical fate and transport, and bioaccumulation as decision‐making tools (USEPA, 2005) and maintains a library of relevant model frameworks (https://www.epa.gov/ceam). Such models have supported decision‐making at contaminated sediment sites across the United States under Superfund, Resource Conservation and Recovery Act, and Clean Water Act (Total Maximum Daily Load) regulatory requirements. Examples include the Upper Hudson River, New York (Connolly et al., 2000; QEA, 1999; USEPA, 2000); the Housatonic River, Massachusetts (USEPA, 2006); the Grasse River, New York (Alcoa, 2012); the Fox River, Wisconsin (RETEC, 2002); the Lower Duwamish Waterway, Washington (LDWG, 2010); the Ports of Los Angeles and Long Beach, California (Anchor QEA, 2017); and the Lower Passaic River, New Jersey (USEPA, 2016).

Nonetheless, the degree to which mathematical modeling factors into remedial decision‐making varies. At some sites, modeling forms an integral component of remedial planning, whereas at other sites, the application of modeling is marginalized and decisions are based on lines of support, such as qualitative estimates of risk reduction based on a conceptual site model or estimates of contaminant mass removal. In response to a survey of state remediation risk management practices conducted by the Interstate Technology and Regulatory Council in 2008, three out of 25 respondents stated that they put significant emphasis on mathematical modeling, 11 put moderate emphasis, nine put some emphasis, and two applied little or no emphasis (ITRC, 2011).

Concerns regarding the use of models include the level of effort, the transactional costs and time required to attain agreement between regulatory agencies and responsible parties, uncertainties associated with model predictions, and limitations in transparency caused by models' complex nature. According to the 2007 National Research Council's report, *Models in Environmental Regulatory Decision Making*, “models will always be constrained by computational limitations, assumptions and knowledge gaps. They can best be viewed as tools to help inform decisions rather than as machines to generate truth or make decisions” (National Research Council, 2007). In a memorandum from the Assistant Administrator of the USEPA Office of Land and Emergency Management to Regional Administrators, Stanislaus concluded:

Future predictions of sediment and fish tissue contaminant concentrations are sometimes presented with a degree of certainty that fails to account for the inherent unknown accuracy of those predictions. Since the accuracy and uncertainty of future projections are generally not known, the use of and comparisons among quantitative endpoints (e.g., time to achieve a sediment or biota contaminant concentration) should be made with a high degree of caution, if at all. (Stanislaus, 2017)

Clearly, there is a need for retrospective studies of the accuracy of model predictions, that is, post‐audits, to evaluate the utility of models in supporting decision‐making (USEPA, 2009). There are a few post‐audits of contaminated sediment site models (Farley & Thomann, 1995; Greenberg et al., 2019; Kreis et al., 2011; Magar et al., 2009; Mossman & Schnoor, 1989; Quadrini et al., 2017; Reidy et al., 2019; Stivers & Patmont, 2015; see Supporting Information Section S1 for brief summaries). Some of these studies include projections of natural recovery in the absence of active remediation, and some incorporate the impacts of local remedial actions. Results are mixed; in some cases, the models projected contaminant concentrations with reasonable accuracy, while in others, the models appreciably over‐ or underestimated the post‐audit data. In some instances, such inaccuracies were by design because decision‐makers opted to make conservative assumptions in the models, which led to the model's underpredicting natural recovery in the system. Several sites where intensive modeling was performed do not yet have a sufficiently long record of post‐remediation data for comparison with model projections. Additional examples of model projections of contaminant concentrations are needed, especially in systems subject to substantial remediation, to fully evaluate the utility and inform future applications of simulation models at contaminated sediment sites.

The Neal's Landfill site (Site) near Bloomington, Indiana, provides an example of the use of a mathematical simulation model in the selection of a remedy for a site, including streams with polychlorinated biphenyl (PCB)‐affected sediment, water, and fish. Data are now available for a post‐audit after the completion of a substantial remedial action, 10‐plus years of subsequent water column monitoring, and completion of a five‐year review of post‐remediation fish monitoring program.

## NEAL'S LANDFILL SITE DESCRIPTION AND HISTORY

**Figure 1:**
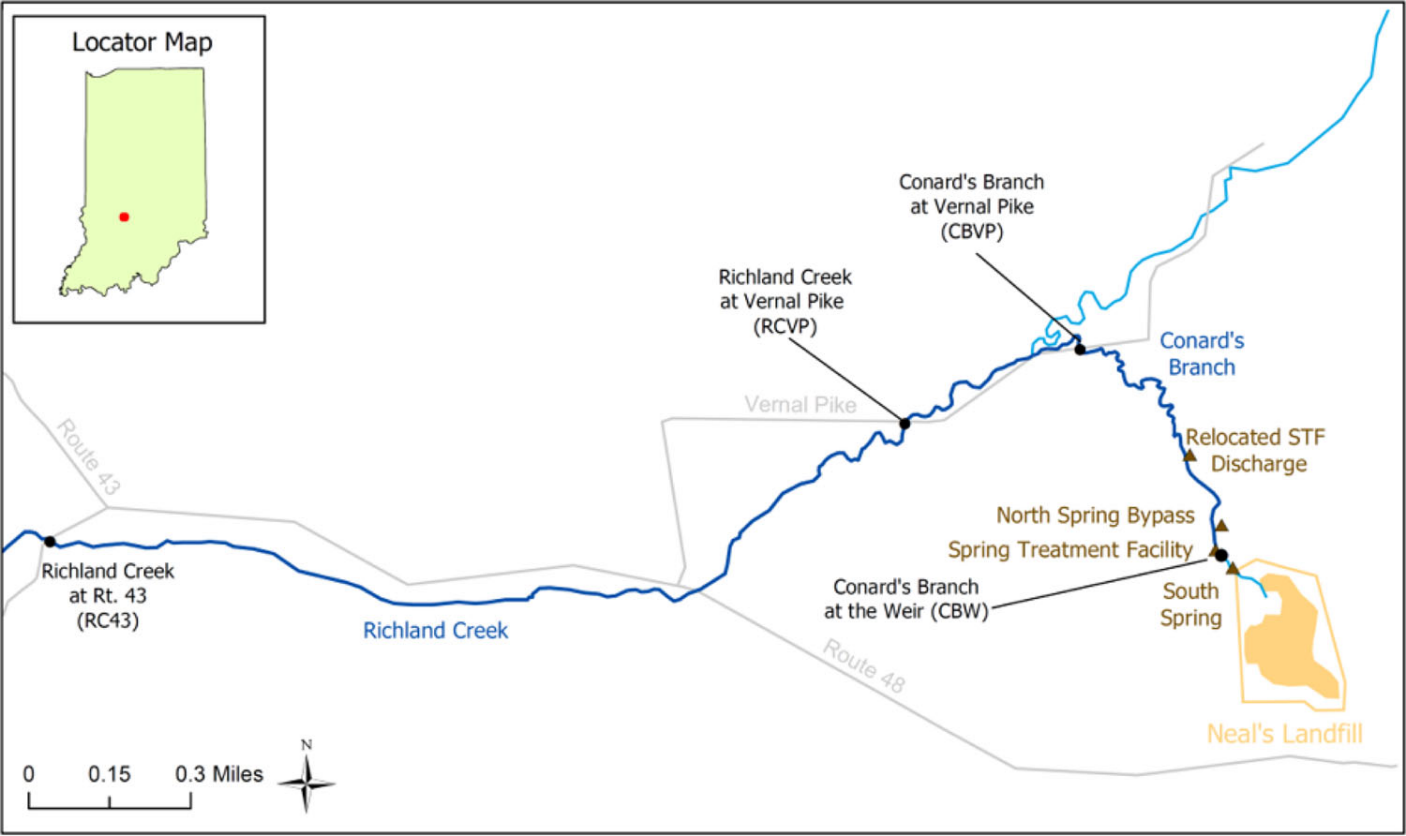
Neal's Landfill site, Indiana, including the landfill, Conard's Branch, and Richland Creek. Sampling locations for water and fish are indicated with black symbols and inflow locations are indicated with brown triangles

Neal's Landfill is an 18‐acre landfill operated from 1950 to 1972 that accepted scrapped electrical capacitors and other materials containing PCBs from a capacitor manufacturing plant in Bloomington, Indiana. The PCBs associated with these wastes were transported to the groundwater beneath the Site, which is underlain by a limestone karst formation containing sinkholes, conduits, and emerging springs. This geologic setting provides a pathway for PCB‐affected groundwater to be transported to springs that emerge near the landfill and flow into Conard's Branch, a small stream situated in the northwest corner of the Site that flows into the larger Richland Creek. Due to the discharge of PCBs associated with spring water flowing into Conard's Branch, PCBs affected the water, sediments, and fish of Conard's Branch and the upper portions of Richland Creek (Figure 1).

Conard's Branch is a small, first‐order, spring‐fed stream, averaging approximately 6 feet in width and 0.6 feet in water depth under base flow conditions. Conard's Branch flows into Richland Creek, which averages approximately 15 feet in width and 0.9 feet in water depth under base flow conditions. Both streams exhibit large fluctuations in flows in response to storm events, with flow rates increasing by up to three orders of magnitude in Conard's Branch and two orders of magnitude in Richland Creek, depending on the size and nature of the storm event. The bottoms of both streams are mainly rocky with intermittent depositional areas (QEA, 2007). The fish community consists largely of creek chub in Conard's Branch, with a more diverse community of approximately 20 species in Richland Creek, including creek chub, longear sunfish, rock bass, and white sucker.

In 1981, the Site was placed on the USEPA Superfund National Priority List. Remedial activities were performed in the 1980s and 1990s, including several actions on the landfill. Sediment removal activities were performed in Conard's Branch and Richland Creek, and a 450‐gallon‐per‐minute (gpm) spring treatment facility (STF) was constructed. The STF collects and treats PCBs in waters flowing from the springs, preventing their flow into Conard's Branch.

Following these initial remedial actions, PCB concentrations in the spring water feeding the stream system declined, and concentrations in Conard's Branch and Richland Creek fish decreased substantially (all analyses and modeling reported here were conducted for total PCBs). Despite these initial reductions, fish PCB concentrations continued to pose potential unacceptable ecological risks to receptors eating fish from the streams. Remaining sources of PCBs to the fish in these streams included the untreated groundwater and spring flows entering Conard's Branch, the STF effluent, the sediments within the streams, and the streambanks. The PCBs associated with the untreated groundwater and spring flows included a portion of the base flow that was not captured and conveyed to the STF for treatment, as well as stormwater flows. During storm conditions, the flow rates could exceed the STF design flow, resulting in short periods during which untreated spring water might have bypassed the STF and flowed directly into Conard's Branch.

It was initially hypothesized that PCB concentrations in fish were strongly influenced by these stormwater inputs, and on this basis, the remedy proposed in the 1990s called for a large storage basin and expanded STF capacity to capture and treat stormwater flows. Subsequently, an investigation was performed that included sampling and analysis of PCBs in surface water (under low‐flow and storm conditions), sediments, and fish in Conard's Branch and Richland Creek, and development of mathematical models incorporating the resulting data. Based on this investigation, alternative remediation plans were proposed.

## ROLE OF MATHEMATICAL MODELING IN REMEDY SELECTION

A chemical fate and transport and bioaccumulation model was developed to quantify the sources of PCBs to fish and, with this information, to predict the effectiveness of alternative remediation scenarios in reducing fish tissue PCB concentrations (QEA, 2007). The model domain included the 0.8‐mile Conard's Branch and a 2.2‐mile stretch of Richland Creek starting at the mouth of Conard's Branch (Figure 1). The results of the model projections formed the basis for the Neal's Landfill elements of the 2007 Record of Decision and the 2009 Agreed Amendment to the Consent Decree for the operable units for groundwater (OU2) and sediment (OU3) at the Site (United States District Court for the Southern District of Indiana Indianapolis Division, 2009), which included:

- Improvement of the spring water collection system to capture PCB‐contaminated groundwater seeps that bypassed the original collection system,
- Installation of a new effluent line further downstream in Conard's Branch to accommodate the new spring water collection system,
- Continued operation of the 450 gpm STF,
- Removal and disposal of instream sediments, bank soils, and floodplain soils in Conard's Branch that exceed cleanup criteria of 1 mg/kg on average for PCBs located in stream sediments and bank soils and 5 mg/kg on average for floodplain soils.

Uncertainty in model predictions was addressed by developing bounding calibrations that used alternative, yet realistic, values of key model parameters, while still honoring the measured surface water and fish concentration data. In addition, a Remedy Confirmation Clause (RCC) was included in the Agreed Amendment to the Consent Decree, requiring evaluation of additional remedial options should fish tissue concentrations not achieve the risk‐based target concentrations within specified time frames.

The OU3 cleanup for instream sediments, bank soils, and floodplain soils in Conard's Branch was completed in 2011 and included removal of more than 900 tons of PCB‐contaminated materials and disposal off site in permitted landfills. Concurrently with the remediation of OU3, construction of the new STF effluent line and improved spring water collection system that makes up the OU2 remediation was completed.

Following the remedy, fish PCB concentrations were monitored to evaluate achievement of the target concentrations specified in the Agreed Amendment to the Consent Decree (Anchor QEA, 2020). These data, along with continued routine monitoring of PCB concentrations in spring and surface waters post‐remedy, provided a unique opportunity to conduct a post‐audit of the model's predictions.

## MODEL DEVELOPMENT

The model framework (i.e., the FORTRAN computer code) used at the Site, AQFATE (Connolly et al., 2000; Mugunthan et al., 2016), is an enhanced version of the Environmental Fluid Dynamics Code (EFDC; Hamrick, 1992) linked to the Water Quality Analysis Simulation for Toxics (WASTOX; Connolly & Winfield, 1984) model. AQFATE has been applied at many contaminated sediment sites. It consists of three submodels (details are provided in QEA, 2007).

1. The hydrodynamic submodel computes flow rates, water depths, current velocities, horizontal mixing, and bed shear stress throughout the model domain. The model solves the three‐dimensional, free‐surface continuity equation and momentum equations, with a barotropic term, a bottom friction term, viscous terms, and advective terms (e.g., Hamrick, 1992; QEA, 1999; Ziegler et al., 2000). A two‐dimensional, vertically averaged configuration was used for this Site.
2. The sediment and PCB fate and transport submodel computes advective and dispersive transport of PCBs and suspended sediments within the water column, deposition and erosion of PCBs and sediments at the bed‐water interface, partitioning of PCBs between the dissolved and particulate phases, and volatilization of PCBs at the air–water interface (e.g., Chapra, 1997; Connolly et al., 2000; Imhoff et al., 2003; Ziegler & Lick, 1986). This submodel also simulates dissolved‐phase exchange of PCBs between sediment porewater and surface water, and particle mixing (i.e., bioturbation) within the sediment bed.
3. The bioenergetics‐based bioaccumulation submodel computes the transfer of PCBs within the food web from sediments and water to the fish species of interest. The model is based on the bioenergetics‐based framework developed by Thomann and Connolly (1984), Connolly (1991), and Connolly et al. (1992). This submodel simulates the uptake of PCBs by diffusion across the gill surface and from food sources, PCB loss by diffusion across the gill, and growth dilution.

These submodels are linked within an integrated framework: Flows, depths, and velocities computed by the hydrodynamic submodel are provided to the sediment transport model, which provides sediment fluxes to the chemical fate and transport model. This in turn provides PCB concentrations in the sediment bed and water column to the bioaccumulation model.

### Hydrodynamics, sediment transport, and chemical fate and transport

The model domain consisted of the full stretch of Conard's Branch from a weir located near the landfill to its mouth at Richland Creek, as well as Richland Creek from the Conard's Branch mouth to the State Route 43 Bridge, a total distance of approximately 3.0 miles (Figure 1). Values for model input parameters were estimated based on site‐specific data, published literature, and models of PCB dynamics developed for other systems. The site‐specific data used to estimate parameter values included:

- Physical data: channel geometry measurements, flow and stage height monitoring, sediment bed thickness mapping, and sediment texture characteristics (i.e., bulk density and moisture content).
- Chemical data: measurement of total suspended solids and PCBs in the water column, and measurements of total organic carbon and PCBs in the sediments.

Final values for a subset of parameters were determined based on calibration of the model to site‐specific field data, including two dye studies performed in 2004, along with stage height, suspended solids, and PCB data for the water column.

For PCBs at the upstream boundary (Conard's Branch Weir), flow‐based statistical relationships, which include a first‐order time decay term to account for a long‐term decline in observed spring PCB concentrations, were developed for base flow and stormwater flow conditions. This decline was statistically significant and was interpreted to reflect source depletion within and beneath the landfill. The PCB inputs to Conard's Branch represented in the model also included the STF effluent (monitored routinely) and additional groundwater bank seeps and spring flow entering in the North Spring area (termed North Spring Bypass [NSB]; see Figure 1).

The model was calibrated to data collected during the period January 2001–December 2005 at three stations (Figure 1): Conard's Branch at Vernal Pike (CBVP), Richland Creek at Vernal Pike (RCVP), and Richland Creek at Route 43 (RC43). These data represented site conditions before the implementation of the remedy specified in the 2007 Record of Decision.

### Bioaccumulation

The bioaccumulation model simulated species of fish that are commonly found in the Site streams: creek chub (a forage fish; Chapman, 2006) in Conard's Branch, and both creek chub and longear sunfish (an omnivorous species; Chapman, 2006) in Richland Creek. The diets of creek chub and longear sunfish consist of mixtures of benthic invertebrates (organisms that accumulate contaminants from the sediment bed), water column invertebrates (organisms that accumulate contaminants from the water column), and terrestrial invertebrates (organisms that were modeled to represent uncontaminated components of the diet). Diet varies seasonally, and was represented in the model (QEA, 2007).

Movement of creek chub and longear sunfish during the warmer months of the year is likely limited, with home ranges less than approximately 100 m (Belica & Rahel, 2008; Berra & Gunning, 1972; Gunning & Shoop, 1963). As marking studies are limited in duration, there is greater uncertainty regarding movement over longer periods, and stream fish may move to deeper waters in winter (Berra & Gunning, 1972).

Growth rates were calculated based on site‐specific age–weight relationships observed in fish. Lipid content used in the model was measured in fish samples collected from the system; values varied seasonally as observed in the data (QEA, 2007). Values for the fish respiration rates and toxicokinetic parameters in the model were based on published literature as well as values used successfully to simulate PCBs at other sites (QEA, 2007). The exposure concentrations in sediment and the water column were computed by the fate and transport model. The model was calibrated to fish tissue data collected during the period January 2001–December 2005.

A complete list of model parameters and their values is presented in QEA (2007).

## MODEL CALIBRATION

The hydrodynamic submodel was calibrated to data collected during two field tracer tests, which permitted estimation of travel times, the amount of dilution from Richland Creek upstream of Conard's Branch, and longitudinal dispersion. In addition, the model was validated against a two‐year record of hourly water surface elevation data from three locations. The ability of the model to accurately predict increases in stage height during elevated flow conditions indicated that the channel slope, mean water depths, and bottom friction were properly represented in the hydrodynamic submodel (QEA, 2007).

**Figure 2:**
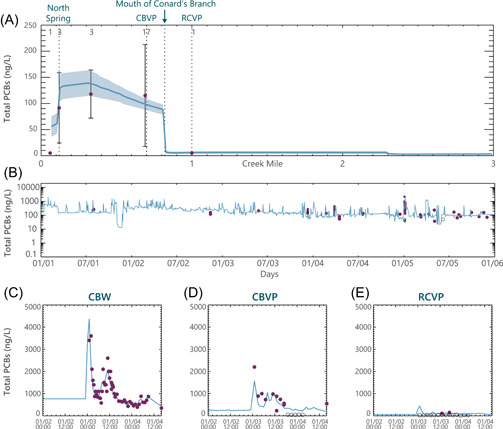
Polychlorinated biphenyl (PCB) concentrations in the water column, model calibration results, and data. (A) Spatial patterns of low‐flow concentrations in Conard's Branch (Mile 0 to 0.8) and Richland Creek (Miles 0.8 to 3), averaged over 2004 and 2005. Lines represent model‐computed mean ± 2 standard errors of the mean (SEM) of daily model calculations for the days on which sampling occurred, for each model grid cell along the center channel. Data displayed as mean ± 2 SEM. The numbers of observations are posted above the error bars. Nondetect polychlorinated biphenyls (PCBs) set at 1/2 method detection limit; number of sampling events posted. (B) Time course of concentrations at Conard's Branch at Vernal Pike (CBVP), 2001 through 2006. (C–E) January 2005 storm event at three locations

The sediment and PCB fate and transport submodel reproduced the spatial pattern in water column PCB concentrations under low‐flow conditions (Figure 2A). This demonstrates that the model captured the increase in PCBs from the key sources along Conard's Branch (i.e., sediments, STF effluent, and the NSB). This also demonstrates the model captured the decreases in PCBs caused by settling of particulate matter (less than 1% of PCB loads to Conard's Branch), volatilization (3% of PCB loads to Conard's Branch), and the large dilution from Richland Creek (approximately a factor of 5–10). A comparison of model‐predicted and observed water column PCB concentrations in Conard's Branch in 2001–2005 illustrates the quality of model calibration over a longer time frame (Figure 2B). In addition, the model‐predicted concentrations of PCBs compared well with high‐frequency data collected at multiple locations during storm events (Figure 2C and E).

**Figure 3:**
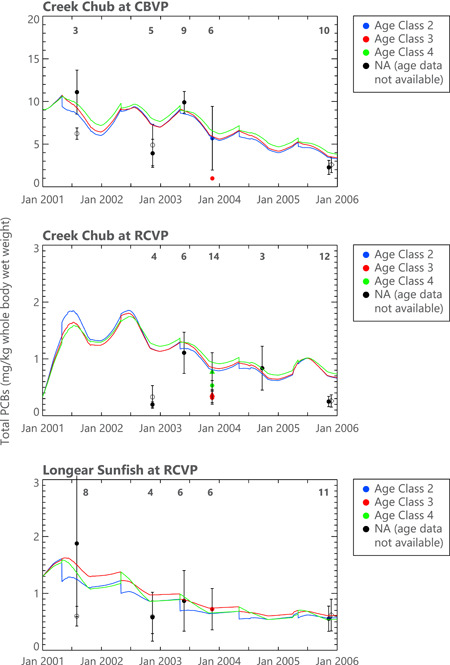
Polychlorinated biphenyl (PCB) concentrations in fish (mg/kg whole body wet weight), model calibration results, and data. Data (symbols; mean ± 2 standard errors of the mean [SEM] and model projections [daily values]). Results presented for the calibration period (2001–2005). Three age classes indicated. Different symbols reflect different laboratories and analytical methods

The bioaccumulation submodel results were compared with five years of measured fish tissue concentrations at the two locations (Figure 3). Conard's Branch fish were collected throughout much of Conard's Branch upstream of the Vernal Pike crossing, although not within 1000 feet of the STF effluent outlet. Richland Creek fish were collected over approximately 2500 feet of creek, downstream of the mouth of Conard's Branch. The coefficient of variation of fish length was generally ca. 10% for each species and location. Samples were combined in composites of three individuals for analysis.

The bioaccumulation submodel captured the long‐term decline. Furthermore, the model captured the seasonal changes in PCB concentrations, which are related to seasonal variations in fish lipid content and diet (both incorporated into the model; QEA, 2007). Finally, the model successfully predicted the lower concentrations found in Richland Creek compared with Conard's Branch, which were caused largely by dilution of Conard's Branch flow in the much larger Richland Creek.

## ASSESSMENT OF PCB SOURCES TO THE WATER COLUMN AND FISH

The contributions of individual sources to PCB concentrations in the water column and fish were estimated with a series of diagnostic simulations conducted with the calibrated model (Table S1). Spring water entering Conard's Branch from upstream was found to be the dominant source to the water column (89%) but contributed only 24–36% of the PCBs in the fish in both streams. This difference arises because much of the load from upstream enters the system under short‐duration storm events, which do not affect the fish as much as the longer duration low‐flow conditions. Thus, although water column PCB concentrations are elevated during high and moderate flow conditions, low‐flow conditions contribute the dominant share of PCBs to the fish. Furthermore, the PCBs that enter Conard's Branch during high‐flow events flow largely through the system and are discharged to Richland Creek because of the short residence time. There is little deposition within Conard's Branch. This limits the long‐term impact of high‐flow events on fish in Conard's Branch. Similarly, deposition in Richland Creek is limited, and storm loads flow largely out of the study area.

These source assessments indicated that stormwater flows, although they contributed a large mass of PCBs to the system, were not greatly significant to fish body burdens. Moreover, although sediments contributed little to the annual PCB load in the Conard's Branch water column, they were important for fish uptake, both directly through feeding in the benthic food web and indirectly through flux of porewater PCBs to the water column. These findings were critical in decision‐making for the Site, as indicated in the projections of the potential benefits of alternative remediation plans.

## PROJECTED IMPACTS OF ALTERNATIVE REMEDIATION PLANS

**Figure 4:**
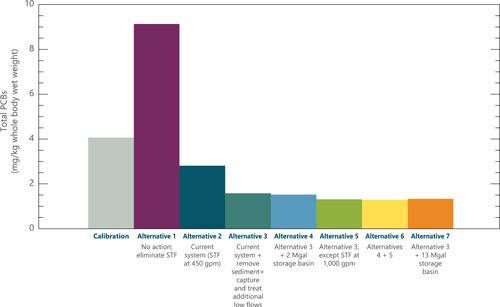
Polychlorinated biphenyl (PCB) concentrations in creek chub at Conard's Branch at Vernal Pike (CBVP) in Conard's Branch, predicted by the model under alternative remedial strategies. Average PCB concentrations in fish tissue from Year 10 of the model projection. STF, spring treatment facility. Alternative 3 was selected

The model was configured to represent seven potential remedial strategies, consisting of varying levels of increased STF treatment and storage capacity, as well as additional source control measures. Details of the model configuration for simulating these alternatives are provided in QEA (2007). Figure 4 presents the average fish PCB concentrations predicted by the model 10 years after remediation as compared with pre‐remediation conditions (as represented by the calibration) for each alternative. Under Alternative 1 (No Action), the STF would be removed. This resulted in higher concentrations in fish than were currently observed. Alternative 2 represented the current system at the time of model development (No Further Action). Concentrations were predicted to decline due to the ongoing recovery of concentrations in spring waters entering Conard's Branch. Alternative 3 included removal of sediment and bank soils along Conard's Branch, the capture of additional NSB flows and routing them to the STF for treatment, and relocation of the STF discharge point (see Figure 1). Alternatives 4–7 included the same elements as Alternative 3, with the addition of other components to provide increased treatment of stormwater flows, including storage basins of varying capacity (up to 13 million gallons) in combination with expanding the STF flow and treatment capacity from the current 450 gpm to rates up to 1000 gpm. The model projections indicated that additional remediation efforts beyond Alternative 3 were predicted to provide little incremental benefit, which is consistent with the aforementioned source assessments that indicated that fish PCB concentrations are driven more by baseflow sources than by stormwater flows. Based on these projections, Alternative 3 was selected by USEPA.

## BOUNDING CALCULATIONS TO QUANTIFY MODEL UNCERTAINTY

To quantitatively assess model uncertainty with respect to its future predictions, a bounding calibration approach was taken; this approach has been recognized by USEPA (2005) as useful for these types of models. Two bounding calibrations were developed, in which the key model parameters were modified within the range of realistic values, while the model continued to compare well with the calibration data (Glaser et al., 2007). In one bounding calibration, parameters were adjusted so that the system would respond to source control actions to a lesser degree than in the base calibration: The natural recovery rate of PCB concentrations in the springs was set at a lower bound based on the monitoring data, PCB loads from NSB were decreased, PCB loads during storms were increased, and, to maintain calibration, PCB loads from sediment to the water column were increased and volatilization rate was adjusted. In the bioaccumulation model, the contribution from the sediment‐based component of the food web was increased and, to maintain calibration, the terrestrial portion of the diet was modified. In the second bounding calibration, the above parameters were adjusted in the opposite directions so that the system would respond to a greater degree than in the base calibration. Model projections were then performed for the base case as well as these two bounding calibrations.

## MODEL POST‐AUDIT

Model projections for Alternative 3 were performed before decision‐making. The original simulations assumed post‐remediation conditions to begin immediately after calibration, starting in 2006. However, remediation was actually completed in 2011. During the six‐year period from the end of calibration to the beginning of the post‐remedy period (2006–2011), PCB concentrations in the incoming spring flows continued the decline that was expected based on data collected during the calibration period, with a first‐order decline rate of approximately 6% per year. Therefore, the post‐remedy surface water concentrations predicted by the model were adjusted by multiplying by 0.70 (based on the 6% decline over the six‐year period, i.e., 1‐e^(−0.06×6)^). The post‐remedy fish concentrations were adjusted by multiplying the spring water and groundwater seepage by the 0.70 factor. However, the STF effluent and sediments were not adjusted because changes in these media were considered to be minor during this period. These adjustments were weighted by their proportional contribution to fish tissue concentrations (Table S1). The final fish multipliers used to account for the six‐year period during which the remedy was designed and constructed were 0.81 (creek chubs at CBVP; see Figure 1), 0.83 (creek chubs at RCVP), and 0.89 (longear sunfish at RCVP). In this way, the model projections of fish PCB levels incorporate both the natural recovery that was expected before remediation as well as the impacts of remediation.

**Figure 5:**
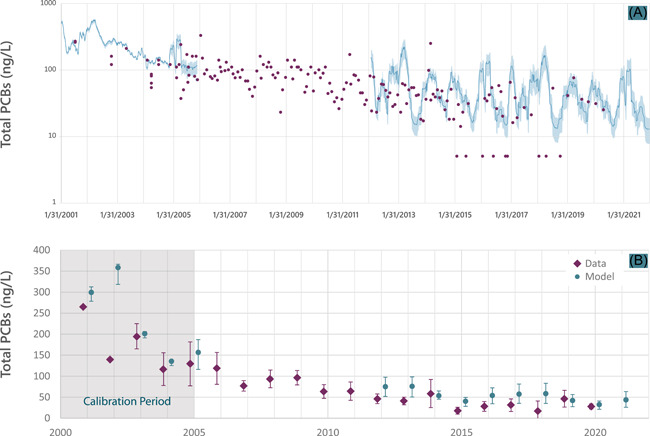
Surface water polychlorinated biphenyl (PCB) concentrations (ng/L) at Conard's Branch at Vernal Pike (CBVP). Data (symbols; individual samples collected during routine [nonstorm] monitoring) and model projections. Results presented for the calibration period (2001–2005) and the projection period (2012–2021). Model projection is for Alternative 3, the selected plan. (A) Model: 120‐day moving average, including base case and lower and upper bound computations (displayed in shading) compared with individual datapoints. (B) Model: base case (symbol) and lower and upper bound computations (error bars) compared with annual average mean ± 2 standard errors of the mean (SEM) for data

**Figure 6:**
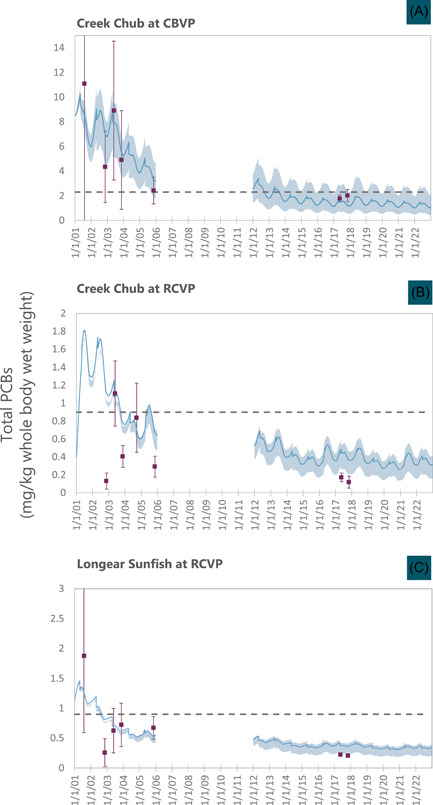
Polychlorinated biphenyl (PCB) concentrations in fish (mg/kg whole body wet weight) at three location and species combinations. Data (symbols; mean ± 2 standard errors of the mean [SEM] and model projections [daily values, including base case and lower and upper bound computations]). Results presented for the calibration period (2001–2005) and the projection period (2012–2021). Model projection is for Alternative 3, the selected plan. Dashed lines: post‐remediation target tissue concentrations

Model results for the calibration period and 10‐year projection period (i.e., post‐audit) are shown in Figure 5 (water) and Figure 6 (fish), which also include the calibration and the post‐remedy data. The data are displayed individually (Figure 5A) or represented as the arithmetic average ±2 standard errors of the mean (SEM; Figures 5B and 6). To reflect prediction uncertainty, the model predictions include results from the base case as well as the two bounding calibrations.

In the water column projections, error bars of the predicted and measured concentrations overlapped in seven out of nine years and nearly overlapped in the other two years (Figure 5B). The base case projected water column concentrations were within a factor of 2 of the average measured concentration in seven out of nine years. Furthermore, the data and model exhibited a similar overall decline from pre‐remedy to post‐remedy conditions. For example, the average measured concentrations declined from 120 to 37 ng/L (2004 and 2005, the last two years of calibration, to 2019 and 2020, the last two years of data collection), a 70% decline. The average modeled concentrations for this same period declined from 150 to 37 ng/L, a 74% decline. In addition, the model generally captured the annual cycle of PCB concentrations associated with annual flow variations (Figure 5A).

For creek chub at CBVP (Figure 1), the May and November 2017 data averages were between the model base case and upper bound (Figure 6A; averages based on 30 fish each; 10 laboratory analyses were performed on composites of three fish each). For creek chub at RCVP (Figure 6B; four analyses [12 fish] in May and three analyses [nine fish] in November), the averages of the data were less than the lower bound model projections, but the error bars of the data overlapped the range of the lower bound computed values for 2016–2018. The data are compared with more than one projection year in order to incorporate uncertainty in hydrology, because the projections employed a repeating cycle of the five years of spring flow rates measured during the calibration period. At this location, the model also overestimated the data for three of the five sampling events during calibration. The overprediction by the model during both the calibration and projection periods could have been caused by underestimation of the proportion of uncontaminated terrestrial food in the diet. During calibration, adjustment of the terrestrial component was considered, but it was decided to retain the potential overestimation by the model as a conservative measure given its use to support decision‐making. For longear sunfish at RCVP (Figure 6C), the two analyses (one composite collected in May and one composite collected in November) were within range of the lower bound model projections for 2016–2018.

In summary, the 2017 fish concentrations were between the base case and upper bound model projections at CBVP and similar to the lower bound model projections at RCVP. Overall, the post‐audit demonstrated that the model successfully predicted declines in surface water and fish tissue PCB concentrations over a decade, including those associated with longer term natural recovery processes and response to an extensive remedial action.

## USE OF THE MODEL IN DECISION‐MAKING

### Consent Decree Remedy Confirmation Clause

Based on the model projections, the USEPA selected Alternative 3 as the remedy to be undertaken (United States District Court for the Southern District of Indiana Indianapolis Division, 2009; USEPA, 2007). However, uncertainties in the model calibration, in future conditions (especially flow rates and rates of recovery of groundwater and spring PCB concentrations), in fish diet, and in monitoring data led to the inclusion of an RCC in the Agreed Amendment to the Consent Decree. The RCC specified target PCB concentrations in the target fish species as arithmetic average PCB concentrations of 2.3 mg/kg whole body wet weight at the Conard's Branch location CBVP (based on the ecological risk assessment; Chapman, 2006), 0.9 mg/kg whole body wet weight at the Richland Creek location RCVP (Chapman, 2006), and 0.2 mg/kg fillet wet weight at the Richland Creek location RC43 (based on protection of human receptors; Tetra Tech, 2006). The RCC specified sampling and statistical testing to be performed every five years post‐remedy to determine whether target concentrations in fish had been achieved and whether there was evidence of decline from pre‐remedy concentrations as predicted by the models. The statistical tests in the RCC defined three categories of outcomes: success (concentrations less than the target by a statistically significant margin), failure (concentrations greater than the target by a statistically significant margin), and inconclusive. “Success” would end the responsible party's obligations except for ongoing operation and maintenance of the STF and long‐term monitoring. “Failure” would potentially lead to modification of the remedy. “Inconclusive” would require continued enhanced monitoring until the subsequent five‐year review. Table S2 lists the statistical hypotheses that were employed in these tests.

### Site closure

The post‐construction average fish PCB concentrations measured in the first five‐year review conducted in 2017 were lower than the target concentrations at all three sampling locations, with *p*‐values associated with Student's *t*‐tests all less than 0.05 (Table S3). On this basis, the implemented remedy was successful, USEPA concluding that no modification of the remedy was needed (USEPA, 2020). Table S3 also provides the average model‐predicted concentrations for 2016–2018 for all three model simulations (base case as well as upper and lower bound alternative calibrations). The dataset is considered to provide a robust estimate of the average fish concentration for 2017: The total number of fish included in each average were 60 (CBVP), 60 (RCVP), and 54 (RC43). Consistent with the requirements of the Agreed Amendment to the Consent Decree, CBVP statistics were based on 100% creek chubs, RCVP statistics were based on approximately equal proportions of top predators, omnivores, and bottom feeders, and RC43 statistics were based on approximately 75% top predators and 25% bottom feeders. Fish sampling will continue at a reduced level, which will permit continued evaluation of the remedy.

In summary, the model predicted that a remedy focused on addressing the PCB sources that contributed most substantially to fish PCB concentrations—remediating stream sediments and capturing and treating baseflow PCB sources (i.e., NSB)—would be sufficient to achieve risk‐based goals. The model also predicted that the costlier collection and treatment of storm flows being considered earlier would have had a minimal incremental reduction in PCB uptake by fish and was not necessary to meet remedial objectives at the Site, that is, the model projection was validated and, on this basis, the remedy was successful.

## MANAGEMENT IMPLICATIONS

This study demonstrates the usefulness of a mechanistic computer simulation model to guide remedial decision‐making at a contaminated sediment site. The combined fate and transport and bioaccumulation model predicted that an alternative less extensive than the treatment of stormwater flows would be sufficient to meet risk‐based goals. The uncertainty analysis was an important component of the project: At CBVP, base case simulations projected fish concentrations less than the target, whereas more conservative upper bound simulations indicated that concentrations might not meet the target (Figure 6). Thus, there was a potential that the selected remedy may not have resulted in fish concentrations that met the target. In contrast, at RCVP, upper bound simulations projected fish concentrations considerably less than the target, and this was observed. This case study demonstrates the usefulness of models in decision‐making for contaminated sediment sites when placed in the context of a decision framework that recognizes and incorporates model uncertainty.

The benefits of a model such as the one reported here extend beyond selecting a remedy. Quantitative models can be used to set realistic expectations regarding the limits of remediation benefits and can aid in interpreting monitoring data and thus in supporting a realistic assessment of remedy success. A computer model is a quantitative representation of the conceptual site model that maintains mass balance and honors site conditions, including not only spatial and temporal patterns in contaminant concentrations but also important ancillary information such as flow rates, total suspended solids concentrations, fish diet, and migration patterns. Moreover, because mechanistic models have now been developed for several decades, they provide confidence that the representation of key processes is consistent with the body of experience at similar sites with similar chemicals. Thus, models provide more than just quantitative projections: They provide a tool to integrate key information available about site contamination and the underlying processes that affect how the contaminants may change in future. Quantitative models come in many forms, ranging from mechanistic simulation models as described here to simple steady state mass balances or statistical relationships. What is critical is to match the right modeling tool to the questions that need to be answered by decision‐makers. At sites with complex hydrodynamics, complex migratory patterns, or multiple contaminant sources, or at sites with insufficient data regarding key underlying processes (e.g., flow, solids loading, and deposition rates), simulation models may be too uncertain to distinguish the relative benefits of remedial alternatives. At such sites, decision‐making must rest on the conceptual site model, with only limited ability to quantitatively distinguish between the long‐term effectiveness of alternative remedies. Even in such situations, simulation models can be deployed diagnostically to explore knowledge gaps and guide additional focused data collection to reduce decision uncertainty.

## CONFLICT OF INTEREST

The authors declare that there are no conflicts of interest.

## Supporting information

Historical review of model post‐audits, additional tables regarding model results and interpretation.

## Data Availability

The data used in model development are available upon request from corresponding author David Glaser (dglaser@anchorqea.com).
