# Supplementary material for: Model‐supported decision‐making at a contaminated sediment site: Post‐audit and site closure
Source: Integr Environ Assess Manag. 2021 Dec 20;18(5):1233–45. doi: 10.1002/ieam.4556 (PMC9543321; doi:10.1002/ieam.4556)
Supplement: Supplementary file 1 — Historical review of model post‐audits, additional tables regarding model results and interpretation. [file IEAM-18-1233-s001.docx]

**Title:  Model-Supported Decision-Making at a Contaminated Sediment Site: Post-Audit and Site Closure Supplemental Data**

Section S1. Brief Summaries of Model Post-Audits

This section provides brief summaries of documented post-audits of contaminated sediment site models.

Schnoor (1981) developed a fate and transport and bioaccumulation model of dieldrin in Coralville Reservoir based on data collected from 1968 to 1978. Mossman and Schnoor (1989) used that model to project fish tissue concentrations through 1987 based on the rate of decline as observed in the original data used to develop the model. Projected concentrations were found to be similar to data collected over that period.

Kreis et al. (2011) post-audited a model of PCB fate and transport and bioaccumulation in Lake Michigan (USEPA, 2006), concluding that over a period of about 15 years, observed and forecasted concentrations were in reasonable agreement.

Farley and Thomann (1995) post-audited a fate and transport and bioaccumulation model of PCBs in the Lower Hudson River that was originally developed in 1987. They concluded that the model-predicted striped bass tissue concentrations compared well with data.

Quadrini et al. (2017) developed a simulation model that included hydrodynamics, sediment transport, chemical fate and transport and bioaccumulation to project PCB concentrations in fish in the Grasse River, New York. It was calibrated to data collected prior to 2002. The model successfully projected the natural recovery observed in annual fish tissue data collected between 2003 through 2015, despite significant external perturbations to the system that the model was not designed to capture.

Reidy et al. (2019) developed a one-dimensional model of mercury concentrations in the surface sediments of the profundal zone of Onondaga Lake, New York. The model projected surface sediment mercury concentrations through 2028 and, consistent with the data, exhibited a decline. This period included extensive littoral zone sediment remediation (between 2012 and 2016), and the model accounted for the impacts of this remediation on the recovery of profundal sediments. Nonetheless, the model overestimated post-remediation surface sediment concentrations. Subsequent studies indicated that this was likely due to the choice of conservative input parameters by decision makers.

A model of PCB fate and transport and bioaccumulation in the Upper Hudson River was developed by the U.S. Environmental Protection Agency (USEPA) and used in support of the 2002 Record of Decision (USEPA, 2002). The projections computed by this model were compared with initial post-remedy data, but the data were deemed insufficient to judge the model (USEPA, 2017; Greenberg et al., 2019). USEPA (2017) concluded that the initial model-data comparisons were generally reasonable, while Field et al. (2016) concluded the model overestimated the rate of recovery.

Magar et al. (2009) and Stivers and Patmont (2015) reviewed case studies of monitored natural recovery at contaminated sediment sites, several of which also included active remediation in portions of the site. Stivers and Patmont (2015) concluded that in some cases, recovery proceeded slower than expected, likely related to the presence of unaccounted for ongoing sources and to temporary impacts of sediment dredging. In some cases, recovery proceeded faster than expected, likely due in part to conservative assumptions used in projecting recovery and to uncertainty in recovery processes.

References

Farley, K. J., & Thomann, R. V. (1995). *An integrated model for the fate and bioaccumulation of PCBs in the Hudson River estuary*. United States. https://www.osti.gov/biblio/458335.

Field, L. J., Kern, J. W., & Rosman, L. (2016). Re-visiting projections of PCBs in Lower Hudson River fish using model emulation. *Science of the Total Environment,* **2016 Jul 1**, 557-558:489–501.

Greenberg, M. S., Traynor, M., Kern, J. W., von Stackelberg, K., & Klawinski, G. (2019). PCBs in Fish Tissues at the Hudson River PCBs Superfund Site: Results of Remedial Action and Early Post-Construction Monitoring. *Battelle Sediments Conference*.

Kreis, R. G., Zhang, X., Murphy, E., Rygwelski, K. R., Warren, G., Horvatin, P. J., Melendez, W., Beck, S. J., & Holsen, T. M. (2011). Post-Audit of Lake Michigan Lake Trout PCB Forecasts. *Lake Michigan: State of the Lake Conference*, September 26 to 28, 2011 Michigan City, Indiana.

Magar, V. S., Chadwick, D. B., Bridges, T. S., Fuchsman, P. C., Conder, J. M., Dekker, T. J., Steevens, J. A., Gustavson, K. E., & Mills, M. A. (2009). *Monitored Natural Recovery at Contaminated Sediment Sites.* Environmental Security Technology Certification Program. ESTCP Project ER-0622.

Mossman, D. J., & Schnoor, J. L. (1989). Post-audit study of dieldrin bioconcentration model. *Journal of Environmental Engineering,* **115**(3), 675–679.

Quadrini, J. D., Connolly, J. P., Constant, A., & McShea, L. (2017). Considerations for Improving the Reliability of Bioaccumulation Model Forecasts: Grasse River Case Study. *Battelle Ninth International Conference on Remediation and Management of Contaminated Sediments*, January 9 to 12, 2017.

Reidy. D., Russell, K. R., Glaza, E., Arrigo, M., Burnham, A., Hague, B., & McAuliffe, J. (2019). MNR at Onondaga Lake: Good Things Come to Those Who Wait. *Battelle Tenth International Conference on Remediation and Management of Contaminated Sediments*. February 11 to 14, 2019. New Orleans, Louisiana.

Schnoor, J. L. (1981). Fate and Transport of Dieldrin in Coralville Reservoir: Residues in Fish and Water Following a Pesticide Ban. *Science*, **211**(4484), 840–842.

Stivers, C., & Patmont, C. (2015). Sediment Monitored Natural Recovery Case Studies. *Battelle Eighth International Conference on Remediation and Management of Contaminated Sediments*, January 12 to 15, 2015.

USEPA (U.S. Environmental Protection Agency). (2002). Hudson River PCBs Site New York Record of Decision.

USEPA. (2006). *Results of the Lake Michigan Mass Balance Project: Polychlorinated Biphenyls Modeling Report*. (EPA-600/R-04/167). Prepared for U.S. Environmental Protection Agency, Great Lakes National Program Office. Prepared by U.S. Environmental Protection Agency Office of Research and Development, National Health and Environmental Effects Research Laboratory, Mid-Continent Ecology Division, Large Lakes and Rivers Forecasting Research Branch Large Lakes Research Station. https://www.epa.gov/greatlakes/lake-michigan-mass-balance-results-and-publications#:~:text=The%20Lake%20Michigan%20Mass%20Balance,
baseline%20to%20gauge%20future%20progress.

USEPA. (2017). *Final Second Five-Year Review Report for Hudson River PCBs Superfund Site*. <https://www.epa.gov/sites/production/files/2019-04/documents/hudson_final_second_five-year_review_report.pdf>.

Section S2. Additional Tables

Table S1
Percent Contributions of Each PCB Source to PCB Concentrations in the Water Column and Fish Computed by the Calibrated Model

| **Source** | **Average Annual PCB Load Entering Conard’s Branch** | **Creek Chubs in Conard’s Branch (CBVP)** | **Creek Chubs in Richland Creek (RCVP)** | **Longear Sunfish in Richland Creek (RCVP)** |
| --- | --- | --- | --- | --- |
| Spring water entering Conard’s Branch from upstream | 89% | 24% | 36% | 24% |
| STF effluent | 4% | 11% | 8% | 6% |
| North Spring and nearby groundwater seepage | 6% | 37% | 21% | 14% |
| Sediments | 1% | 27% | 35% | 56% |

Notes:

CBVP: Conard’s Branch at Vernal Pike

RCVP: Richland Creek at Vernal Pike

STF: spring treatment facility

Table S2
Remedy Confirmation Clause Hypothesis Tests

| **Test No.** | **Frequency** | **Hypotheses** | **Interpretation** |
| --- | --- | --- | --- |
| 1 | Every 5 years | H_0_: fish data >= target  HA: fish data <  target | If H_0_ rejected, remedy successful |
| 2 | Every 5 years | H_0_: fish data <= target  H_A_: fish data >  target | If H_0_ rejected, pass to Test 3 |
| 3 | First 5-year event, if H_0_ rejected in Test 2 | H_0_: fish data <= pre-remedy concentration  H_A_: fish data > pre-remedy concentration | If H_0_ rejected, remedy failure |
| 4 | Second 5-year event, if H_0_ rejected in Test 2 | H_0_: no downwards trend in fish data  H_A_: downwards trend in fish data | If H_0_ is not rejected, then conclude remedy failure |

Table S3
Post-Remedy Hypothesis Test Results

| **Location** | **Target Concentration  (mg/kg wet weight)** | **Concentration Measured in 2017 mean +/- 2 SEM (n)** | **P value  (Student’s t-test)** | **Average Model-Predicted Concentration 2016 to 2018  (best estimate and bounding cases)** |
| --- | --- | --- | --- | --- |
| CBVP | 2.3 (whole body) | 1.9 +/- 0.40 (20) | 0.005 | 1.5 (0.69 – 2.7) |
| RCVP | 0.9 (whole body) | 0.24 +/- 0.10 (20) | <0.001 | Creek chub: 0.42 (0.24 – 0.51)  Longear sunfish: 0.38 (0.24 – 0.43) |
| RC43 | 0.2 (fillet) | 0.047 +/- 0.030 (18) | <0.001 |  |

Notes:

CBVP: Conard’s Branch at Vernal Pike

mg/kg: milligram per kilogram

RC43: Richland Creek at Route 43

RCVP: Richland Creek at Vernal Pike

SEM: Standard Error of the Mean

n: number of composite samples. Three fish were included in each composite; thus, the number of fish included in each average is three times the posted value.

The measured concentrations represent species mixtures as specified in in the Agreed Amendment to the Consent Decree. CBVP statistics were based on 100% creek chubs, RCVP statistics were based on approximately equal proportions of top predators, omnivores, and bottom feeders. RC43 statistics were based on approximately 75% top predators and 25% bottom feeders.
